# A Novel Si_2_Ga_2_S_2_ Monolayer for Photovoltaic Applications

**DOI:** 10.1021/acsomega.5c01810

**Published:** 2025-05-12

**Authors:** Willian O. Santos, Leonardo S. Barbosa, Edvan Moreira, David L. Azevedo

**Affiliations:** † Postgraduate Program in Physics, 74391 Federal University of Sergipe (UFS), Cidade Univ. Prof. José Aloísio de Campos, 49107-230 São Cristóvão, Sergipe, Brazil; ‡ Postgraduate Program in Aerospace Engineering, 119484 State University of Maranhão (UEMA), Cidade Universitária Paulo VI, 65055-310 São Luís, Maranhão, Brazil; § Department of Physics, State University of Maranhão (UEMA), Cidade Universitária Paulo VI, 65055-310 São Luís, Maranhão, Brazil; ∥ Institute of Physics, University of Brasilia (UnB), Campus Universitário Darcy Ribeiro—Asa Norte, 70919-970 Brasília, Distrito Federal, Brazil

## Abstract

Two-dimensional materials have shown potential applications ranging from electronic devices to solar panels as their properties significantly differ from those of bulk materials. Herein, density functional theory is used to investigate the structural, electronic, optical, power conversion efficiency and Shockley–Queisser limit, and thermodynamic properties, as well as the energetic stability and dispersion phonon of Si_2_Ga_2_S_2_ monolayer. The results show that using approaches based on the generalized gradient approximation (GGA) and the HSE06 hybrid exchange–correlation functional for the minimum energy optimized structure, a direct bandgap of 1.247 and 2.132 eV was obtained within the GGA-Perdew–Burke–Ernzerhof (GGA-PBE) and HSE06 calculation level, respectively. The optical absorption was sensitive to the plane of polarization of the incident light, especially in the UV–vis regions. Power conversion efficiency (PCE) and Shockley–Queisser minimum limit estimated were approximately 19.46%. Moreover, from calculations of the thermodynamic potentials within the PBE functional, the free energy (*F*) indicates that this Si_2_Ga_2_S_2_ monolayer could potentially be synthesized spontaneously at low temperatures. All properties calculated in this study indicate that the Si_2_Ga_2_S_2_ monolayer could have potential applications in solar cells. However, only experiments can confirm this.

## Graphical abstract

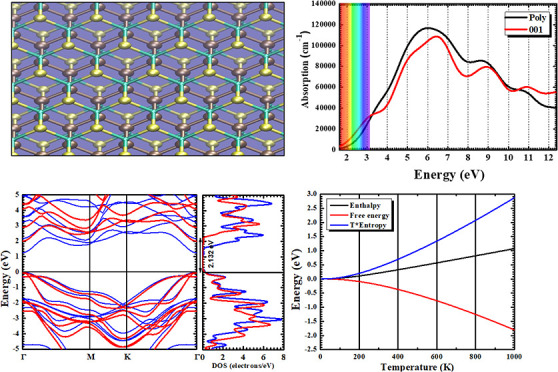

## Introduction

Humanity’s progress has been accompanied by the search for renewable energy sources. Given this, the burning of fossil fuels, which is still the largest source of energy in the world, represents a major setback, as the environmental impacts it causes worsen the greenhouse effect and contribute to the destruction of the ozone layer,^1–3^ melting of polar ice caps, and air, seas, and river pollution,^4,5^ among others, and is a finite resource. The indispensability of environmental preservation generates the need to produce clean energy that causes the least impact possible, and one of the solutions to this issue is the use of energy generated through sunlight, using photovoltaic cells composed of silicon, an abundant material on the planet.^6^

Since the synthesis of graphene in 2004,^7–9^ low-dimensional materials have been exhaustively researched due to their promising applications in optoelectronics. Among these materials are the two-dimensional (2D) transition-metal dichalcogenides (TMDs) and perovskites as promising components for solar cell devices due to their unique optoelectronic properties.^10^ Perovskites are attracting more attention as a photovoltaic material due to exhibiting a longer electron/hole diffusion length than silicon, resulting in an improved power conversion efficiency (PCE).^11–14^ Recently, it was reported an improvement in perovskite-Copper Indium Gallium Selenide (CIGS)-based solar cells using the BaSnO_3_ charge transport layer that can deliver a PCE as high as 39%, which can contribute to the high-efficiency solar cell fabrication roadmap.^15^ In addition, a detailed investigation of double perovskite (FA)_2_BiCuI_6_-based perovskite solar cells utilizing diverse kesterites as hole transport layers (HTL) and titanium-based electron transport layers (ETLs) obtained optimized PCE above 25%.^16^ Beyond that, to further improve the device performance of perovskite as solar cells, a series of 2D materials such as graphene, MXenes, TMDs, and others have been introduced into the cell structure with remarkable effects.^17,18^

Also, organic solar cells (OSCs) have seen great progress in PCEs recently. A novel approach to developing high-efficiency and stable OSCs was reported, where a PCE of 19.41% was achieved.^19^ Furthermore, benzodithiophene-based novel donor molecules were investigated by density functional theory (DFT) and a PCE of 19.09% is predicted for organic solar cells.^20^ Recently, other great achievements were reported in OSCs, where a PCE of 19.35% was obtained, showing photostability with PCE decaying by <20% after, approximately, 30 days of continuous irradiation.^21^

TMDs display robust light–matter interaction, photoluminescence,^22^ and exceptional optical performance with promising applications, e.g., for organic and perovskite solar cells playing important roles as electrodes, hole/electron extraction, and interfacial layers.^11^ Furthermore, single-junction solar cells using TMD films as thin as 50 nm can reach up to 25% PCE, making them ideal for high-specific-power photovoltaics.^23^ Scalable methods for large-area WSe_2_ film synthesis for use as absorber layers in ultrathin high-specific-power solar cells were reported,^24^ where WSe_2_ films can provide efficiency up to 22.3% and power over 10× higher than other thin-film solar cells. Beyond that, the TMD heterojunctions MoTe_2_/MoSe_2_ have photoconversion efficiency as high as 25.84% providing theoretical evidence of excellent candidate TMDs for the advancement of optoelectronics.^25^

Silicon-based materials are still the most used in solar cell devices, dominating the global market for solar-generated electricity^26–28^ because of their low bandgap, availability (second most abundant element in the Earth’s crust^29^), nontoxicity, and well-established processing techniques.^11,28^ However, the search for reducing the cost per watt has motivated the investigation of novel silicon-based materials and other types of materials for photovoltaic devices.

Motivated by the possibility of a new 2D material composed of silicon, the objectives of this study are to investigate the structural and electronic properties, optical absorption, phonon dispersion, thermodynamic potentials, and constant volume heat capacity using DFT and density functional perturbation theory (DFPT) calculations of the Si_2_Ga_2_S_2_ structure. However, the Si_2_Ga_2_S_2_ monolayer has not been explored enough and a theoretical DFT study is still lacking. This paper is organized as follows: details of the theoretical method are reported in the Computational Details section. The Results and Discussion section discusses geometry optimization, cohesive energy, phonon dispersion curves, electronic properties, optical absorption, Shockley–Queisser limit, thermodynamic potentials, and constant volume heat capacities. Finally, a summary is presented in the Conclusions section.

## Computational Details

To determine the electronic, optical, PCE, and vibrational characteristics of monolayer Si_2_Ga_2_S_2_, the calculations in this article were performed with the help of first-principles-based DFT techniques.^30,31^ The unit cell of the solid Si_2_Ga_2_S_2_, containing six atoms, was obtained from the open-access Computational 2D Materials Database (C2DB).^32^

The CASTEP^33,34^ package was used to calculate the electronic characteristics of the material.^33^ Bandgap energy calculations were performed using the generalized gradient approximations (GGA) proposed by Perdew–Burke–Ernzerhof (PBE)^35,36^ to describe the exchange and correlation energy of the Si_2_Ga_2_S_2_ compound. Single-point hybrid exchange–correlation functional (HSE06)^37^ calculation from GGA-PBE optimized geometry was also used to better describe the electronic structure, with optimized screening parameters^38^ as recommended for bandgap calculations in the CASTEP code.

Integration in the Brillouin zone was performed through a 4 × 4 × 1 *k*-point sampling using a grid of Monkhorst–Pack,^39^ taking into account the following electronic valence configuration for each atomic element: Si 3s^2^3p^2^, Ga 3d^10^4s^2^4p^1^, and S 3s^2^3p^4^.

A cutoff energy of 800 eV was defined. To guarantee accurate convergence of atomic positions, self-consistent parameter calculations were used with the following criteria of the previous work: total energy change less than 0.10 × 10^–4^ eV/atom, maximum ionic force on each atom below 0.03 eV/Å, pressure less than 0.05 GPa, and maximum atomic displacement not exceeding 0.10 × 10^–2^ Å. These calculations were performed using the Broyden–Fletcher–Goldfarb–Shanno (BFGS) minimizer.^40^ Additionally, vibrational property calculations were carried out using density functional perturbation theory (DFPT), implemented within the linear response formalism.^41,42^

In Figure 1, an illustration of the unit cell of the Si_2_Ga_2_S_2_ nanostructure is shown with different perspectives, where the green spheres represent the sulfur atoms, yellow spheres represent the silicon atoms, and brown spheres represent the gallium atoms, which contain two silicon (Si), two gallium (Ga) atoms, and two sulfur (S).

**Figure 1:**
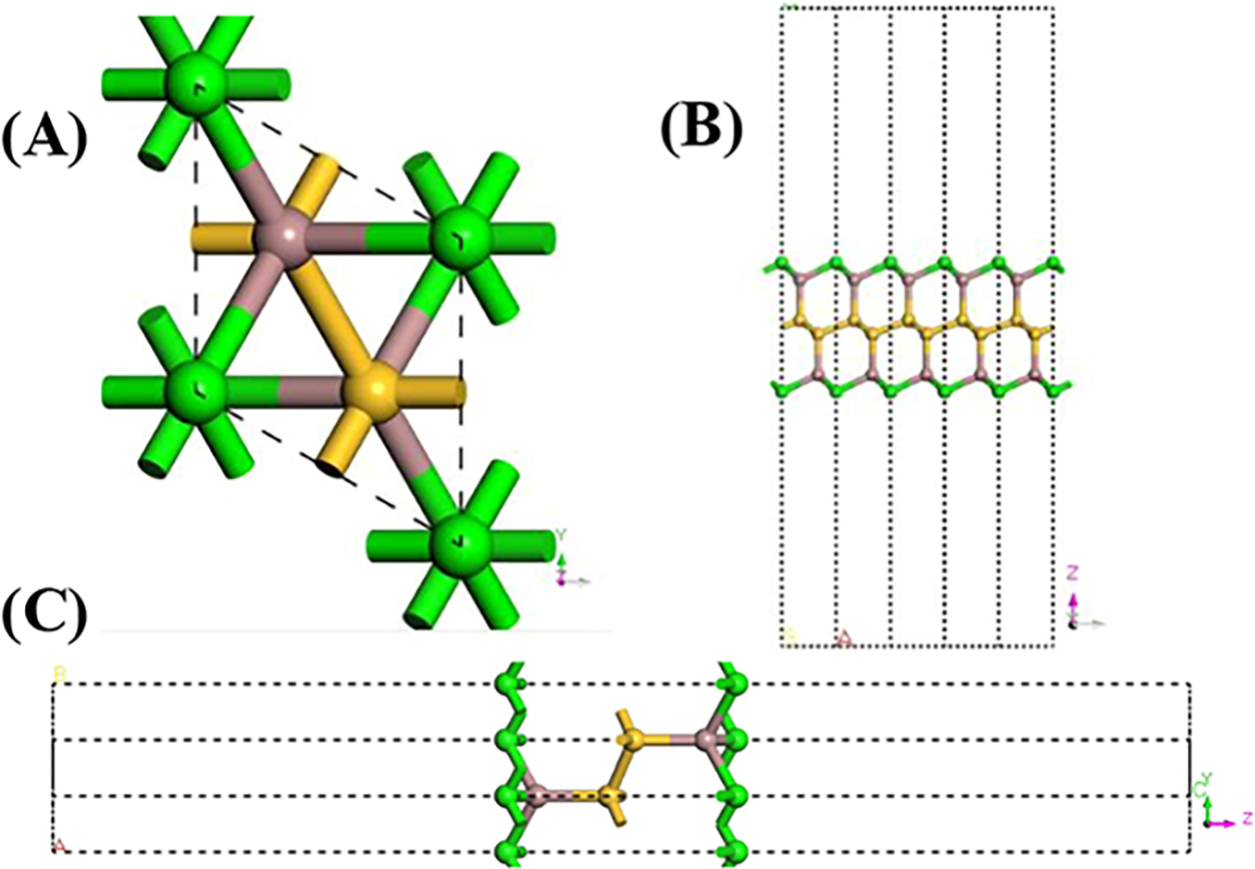
Crystalline structure of Si_2_Ga_2_S_2_ with different perspectives of the primitive cell and atomic labels. (A) Primitive cell. (B) Cell replicated in the *A* direction. (C) Cell replicated in the *B* direction.

The monolayer of Si_2_Ga_2_S_2_ is a structure that crystallizes in a Bravais^43^ lattice of trigonal symmetry, belonging to group 164 (*P* 3 *®m*1), with the crystal lattice parameters *a* = *b* = 3.752 Å and *c* = 38.171 Å and primitive cell volume *V* = 465.439 Å^3^, which is similar to other silicon-based monolayers previously reported in the literature.^44–47^

## Results and Discussion

### Stability Analysis and Phonon Dispersion

To study the dynamic stability of the Si_2_Ga_2_S_2_ monolayer, the cohesive energy and phonon dispersion were calculated. The results are shown in Table 1 and Figure 2. Table 1 shows the energy values obtained in the process of optimizing the geometry of the Si_2_Ga_2_S_2_ monolayer, and Figure 2 shows the vibrational spectrum.

**Figure 2:**
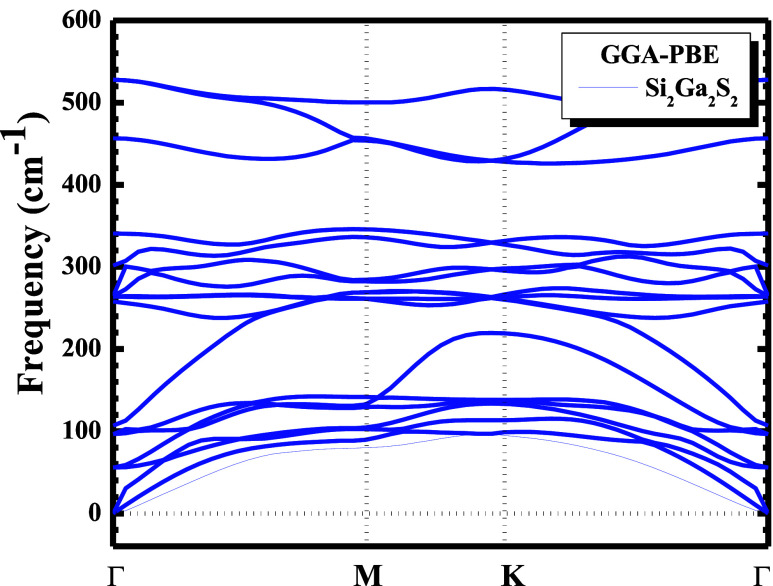
Phonon dispersion of the Si_2_Ga_2_S_2_ structure, calculated using the GGA-PBE correlation–exchange functional for frequencies ranging from 0 to 600 cm^–1^.

**Table 1:** Energies Were Calculated Using the DFT/GGA-PBE Method for Systems and Single Atoms

atomic energies	energy (eV)
*E*_Si_	–212.664
*E*_Ga_	–3418.855
*E*_S_	–549.208
*E*_Si_2_Ga_2_S_2__	–4193.084

Thus, to investigate the energetic stability of the Si_2_Ga_2_S_2_ monolayer, the cohesive energy is calculated (*E*_coh_),^48,49^ using the data in Table 1 with the following expression:

1 $$E_{\mathrm{coh}}=\frac{\sum E_{\mathrm{isolated}}-E_{\mathrm{monolayer}}}{N}$$

where *E*_isolated_ is the energies of the isolated Si, Ga, and S atoms, respectively. *E*_monolayer_ is the total energy of the unit cell of each monolayer. The estimated value for cohesive energy using eq 1 is approximately 2.05 eV/atom, which is in agreement with data found in the literature,^50–53^ indicating that the investigated systems are energetically stable.

Subsequently, the phonon dispersion was calculated using a linear response^54^ implemented in the CASTEP code.^34^ The phonon dispersion can be described using a harmonic approximation based on the knowledge of just one fundamental quantity, the force constants matrix:

2 $$D_{\mu \nu }\left(q\right)=\frac{1}{N_{R}}\sum_{R}D_{\mu \nu }\left(q\right)\exp\left(-i\mathrm{qR}\right)$$

Equation 2 shows a classical representation for the description of motion in matrix language in such a way that the atomic displacement suffered by the perturbation is described in the form of plane waves:

3 $$u\left(R,t\right)=\varepsilon \;e^{i\left[\mathrm{qR}-\omega \left(q\right)t\right]}$$

where ω is the wave frequency and *q* is the wave number, which in this case, *q* = 2π/λ.

The phonon dispersion curves along the high-symmetry points in the Brillouin zone of Si_2_Ga_2_S_2_, showing the frequency range from 0 to 600 cm^–1^, is presented in Figure 2. The modes that vary from Γ to Γ, observed in the phonon spectrum, are related to the acoustic vibrations of the material, while the others are related to the optical modes. The estimated values of the cohesive energy and the phonon spectrum (Figure 2) of Si_2_Ga_2_S_2_ suggest that the system is stable and can be synthesized.

### Electronic Properties

Figure 3 represents the Kohn–Sham electronic band structure and density of states (DOS) of monolayer Si_2_Ga_2_S_2_. Eigenenergies were calculated based on the correlation–exchange potential using the GGA-PBE functional, which gives accurate information about the shape of the energy bands (such as valence band maximum (VBM) and conduction band minimum (CBM)). However, the material’s bandgap is significantly underestimated, so HSE06 single-point hybrid functional calculation was employed for bandgap correction.

**Figure 3:**
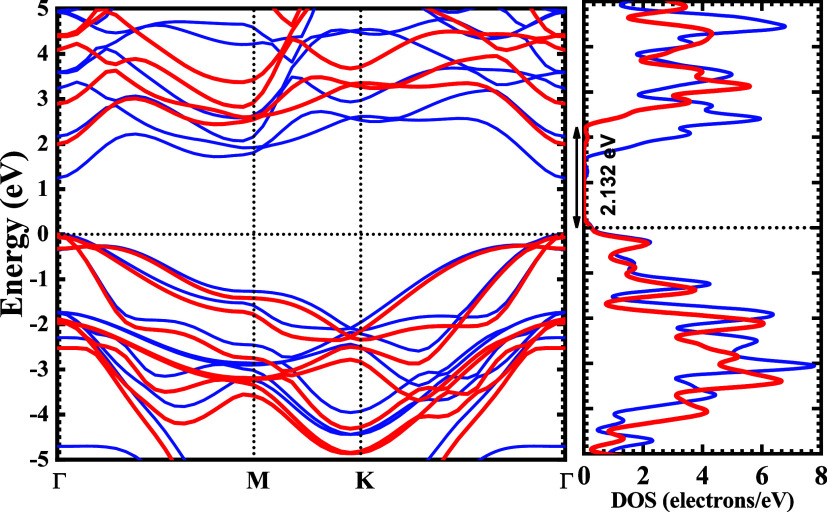
Kohn–Sham electronic band structure and total density of states (DOS) for Si_2_Ga_2_S_2_ (blue solid line) calculated using the GGA-PBE exchange–correlation functional and the HSE06 hybrid exchange–correlation functional (red solid line). (For interpretation of the references to colors in this figure legend, the reader is referred to the web version of this paper).

Figure 3 shows the eigenvalues obtained through solutions of the electronic Kohn–Sham equations, which have two defined regions. It is observed that the eigenenergies related to the valence band (VB) vary from −5 to 0 eV and the conduction band (CB) from 0 to 5 eV. The Fermi level (ε_F_) (horizontal dotted black line) was set to 0 eV, thus ensuring the highest energy for a valence electron. It is also noticed that, for the electronic transition to occur using the GGA-PBE-type approach, the minimum value of energy required is 1.247 and 2.132 eV for the HSE06 hybrid approximation; therefore, the Si_2_Ga_2_S_2_ monolayer is a semiconductor with direct bandgap characteristic (Γ → Γ). The bandgap energies for crystalline silicon (Si) are 1.12 eV at 300 K and 1.16 eV at 0 K.^55^ The Si_2_Ga_2_S_2_ monolayer has a higher estimated bandgap energy than crystalline silicon. However, the advantage of the Si_2_Ga_2_S_2_ monolayer is its direct bandgap characteristic, which may favor applications in optoelectronics and solar cells, while crystalline silicon has an indirect bandgap.^56^ This semiconductor feature of the Si_2_Ga_2_S_2_ monolayer also indicates the possibility of applications in specific electronic device designs, such as field-effect transistors and nanosensors.

Looking at the DOS curves (Figure 3), the GGA-PBE states are reduced relative to the HSE06 calculation in the region under the Fermi level energy; the most extensive peak is located at −3.05 and −3.39 eV, where there will be a considerable presence of occupied states.

In Figure 4, the partial density of states (PDOS) for Si_2_Ga_2_S_2_ individual atoms is presented. The results show that the most intense contribution of each individual atom (Si, Ga, and S) corresponds to the p-orbital. The p-orbital dominance in the valence band and conduction band can be explained by the electronic structure and bonding characteristics of the material studied. The S p, Si p, and Ga p are the orbitals that most contribute to the valence region (−4 to 0 eV) and the conduction region (2–4 eV). This behavior arises from the electronic structure of the material, where the valence band is primarily composed of S 3p and its states, hybridized with contributions from Ga 4p and Si 3p.

**Figure 4:**
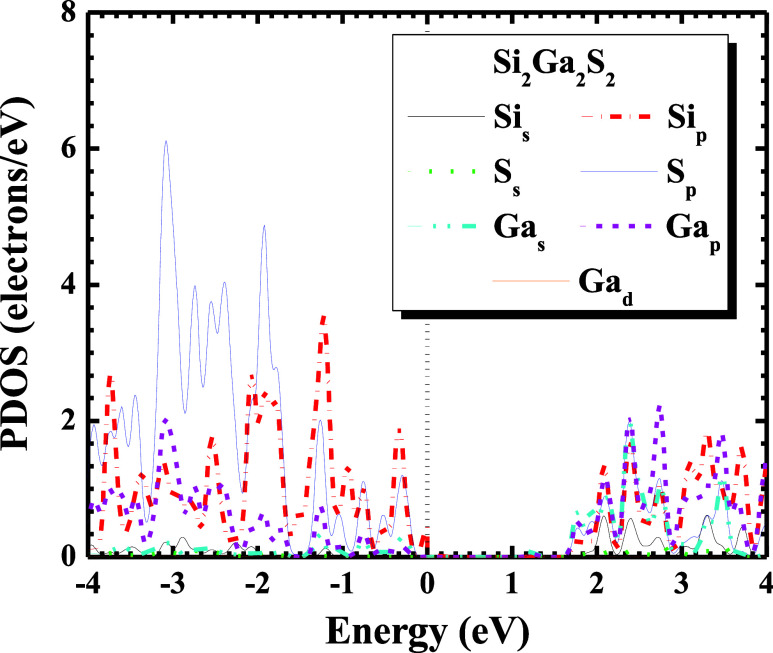
Partial density of states (PDOS) for Si_2_Ga_2_S_2_ individual atoms calculated using only the GGA-PBE exchange–correlation functional. (For interpretation of the references to colors in this figure legend, the reader is referred to the web version of this paper).

### Optical Properties

To obtain the optical absorption spectra of the Si_2_Ga_2_S_2_ monolayer depicted in Figure 5, the GGA-PBE exchange–correlation functional is employed. In Figure 5, it can be observed that the electronic excitations of the Si_2_Ga_2_S_2_ monolayer are related to the direction of the incident radiation. Therefore, this fact occurs when a crystal has the property of optical anisotropy. For all crystallographic directions, the Si_2_Ga_2_S_2_ monolayer presents absorption in the visible and ultraviolet (UV) regions of the electromagnetic spectrum with absorption in the visible (∼1.6–3.0 eV) and the ultraviolet (UV) (∼3.0–10.0 eV). In the visible region of the electromagnetic spectrum, it is discovered that the directions [001], [011], [101], and [111] are predominant for electronic excitations presenting similar behavior. In the UVB region (∼3.9–4.4 eV) and the UVC region (∼4.4–12.4 eV), the most intensity absorption peaks are in the [010], [100], and Poly directions. The crystallographic direction [100] is superimposed on [010]. Just for comparison, the experimental absorption coefficient α (cm^–1^) of crystalline silicon at room temperature in the wavelength range of 500–1000 nm (∼2.4–1.2 eV) varies from 10^4^ to 10^2^ cm^–1^.^57^ A similar behavior is observed in the Si_2_Ga_2_S_2_ monolayer in the [001], [011], [101], and [111] directions. The optical absorption results suggest that the Si_2_Ga_2_S_2_ monolayer is suitable for use in future solar cell devices. Given its intense absorption in the [001] direction in the ultraviolet region, its efficiency can be further increased using multi-junction techniques. Furthermore, due to the high absorption in UV, Si_2_Ga_2_S_2_ monolayer could be used as a UV detector.

**Figure 5:**
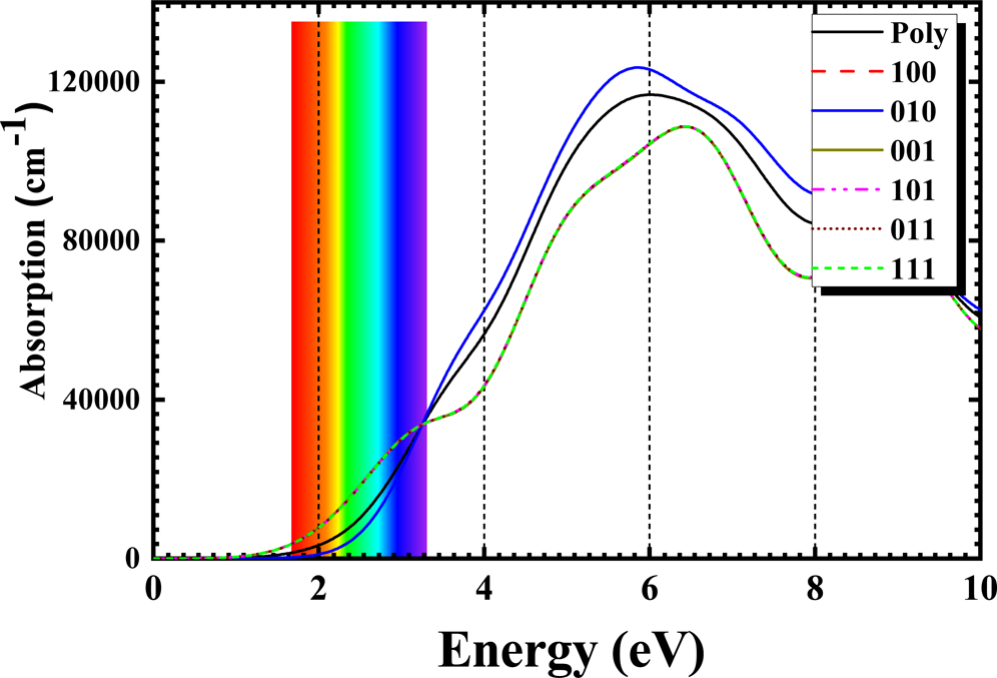
Optical absorption spectra of Si_2_Ga_2_S_2_ as a function of energy (in eV), using the GGA-PBE exchange–correlation functional near the energy bandgap when the incident radiation is polarized along the crystalline planes [001, 010, 100, 101, 110, and 111] and for the polycrystalline sample (Poly).

### Shockley–Queisser Limit

The method is based on the results of Rühle,^58^ where the Shockley–Queisser limit refers to the theoretical maximum (η %) efficiency of a single-junction photovoltaic cell, where the principle is based on determining the electronic bandgap, to stipulate the maximum sunlight to electrical energy conversion efficiency.

In Table 2, the results of the calculated bandgaps of the GGA-PBE functional and the HSE06 hybrid functional are presented, where the values from Table 1 of ref 58 were used to interpolate the PCE η for the Si_2_Ga_2_S_2_ monolayer. In ref 58, the maximum PCE η is 32.74 and 20.50% for 1.2 and 2.1 eV bandgap values, respectively. Through these results, the results of Table 2 are obtained by interpolation. Furthermore, using the results of Table 2, the minimum PCE for the Si_2_Ga_2_S_2_ monolayer is 19.46%, showing potential for applications in photovoltaic cells. Undoubtedly, the multi-junction technique could significantly improve this efficiency. Just for comparison, the best PCE of other Si-based materials is 26%.^59,60^ However, the Si solar cells used nowadays present some drawbacks, for example, relatively weak absorption of sunlight because of the indirect nature of their bandgap.^59^ However, the investigated Si_2_Ga_2_S_2_ monolayer had a direct bandgap, which could increase sunlight absorption, improving its efficiency. TMD solar cells, e.g., MoS_2_, MoSe_2_, WS_2_, and WSe_2_ as thin as 50 nm can achieve up to 25% PCE; however, efforts in TMD solar cell designs must be dedicated to unlocking the potential of TMD materials for high PCE and specific power on a large industrial scale.^23^ The crystalline silicon material is the most important photovoltaic technology nowadays, with a global market share of about 90%,^61^ and will likely remain the most widely used material in photovoltaic cells in the coming years. In this context, a Si_2_Ga_2_S_2_ monolayer can make the difference because it is a Si-based material with direct bandgap semiconductor characteristics with reasonable theoretical PCE.

**Table 2:** Calculated Bandgap Energy (*E*_g_) and PCE Interpolation Values Estimated from the Shockley–Queisser Limit (η %), Considering the Functionals PBE and HSE06

functional	*E*_g_ (eV)	η %
PBE	1.247	32.65
HSE06	2.132	19.46

### Thermodynamic Properties

The result of calculating the phonon spectrum of the Si_2_Ga_2_S_2_ monolayer is equivalent to an increase in its internal energy through crystalline vibrations.^54,62,63^ Thermodynamic potentials were estimated using the Si_2_Ga_2_S_2_ vibrational frequencies obtained in phonon calculations, which were determined using the density functional perturbation theory (DFPT) formalism.^42,64^ In a multielectron system with an average atomic volume *V* at temperature *T* using the quasi-harmonic approximation, the Helmholtz free energy *F*(*V*, *T*) is given by^65,66^

4 $$F\left(V,T\right)=E_{c}\left(V\right)+F_{\mathrm{ph}}\left(V,T\right)+F_{\mathrm{el}}\left(V,T\right)$$

*E*_c_ is the total energy at 0 K, *F*_ph_ is the vibrational free energy of the lattice ions, and *F*_el_ is the thermal electronic contribution to the Helmholtz energy, using the relationship of eq 5:

5 TS=U−F

The thermodynamic potentials, such as enthalpy (*H*), free energy (*F*), and temperature times entropy term (*T* × *S*) are shown in Figure 6.

**Figure 6:**
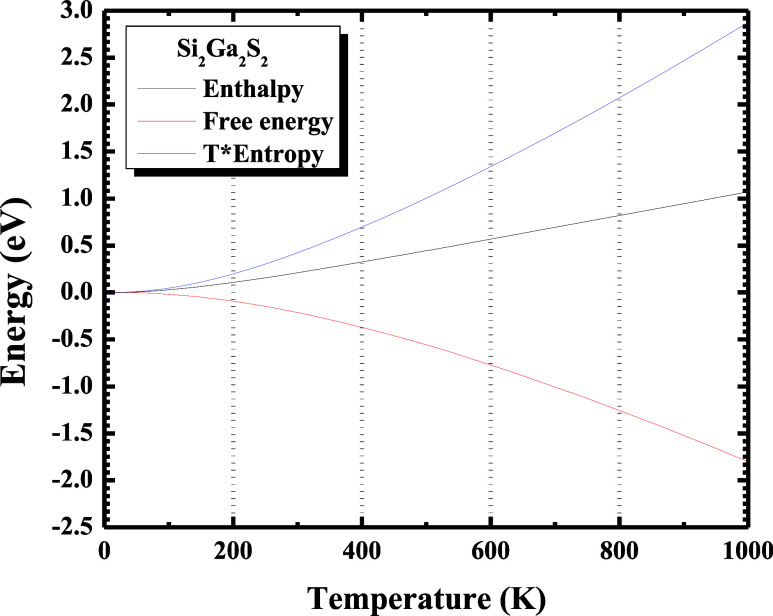
Profiles of the calculated thermodynamic potentials: enthalpy (black line), free energy (red line), and *T* × entropy (blue line) as a function of the temperature for Si_2_Ga_2_S_2_ monolayer. (For interpretation of the references to color in this figure legend, the reader is referred to the web version of this paper).

Figure 6 shows that the function *H*(*T*) has a linear trend with increasing temperature; *F*(*T*) converges to a negative value as the temperature increases, indicating that the synthesis process could be spontaneous in the Si_2_Ga_2_S_2_ monolayer; *T* × *S*(*T*) increases as a function of temperature, which is in accordance with the second law of thermodynamics, where its maximum value is ∼2.8 eV.

The constant volume heat capacity (*C*_V_) is represented in Figure 7 as a function of temperature in K. *C*_V_ increases rapidly as the temperature increases in a range of 0–250 K, reaching the Dulong–Petit limit^43^ (≈29.4 cal/(cell·K)). This result can be used to predict the phase stability of different structural modifications for the Si_2_Ga_2_S_2_ monolayer.

**Figure 7:**
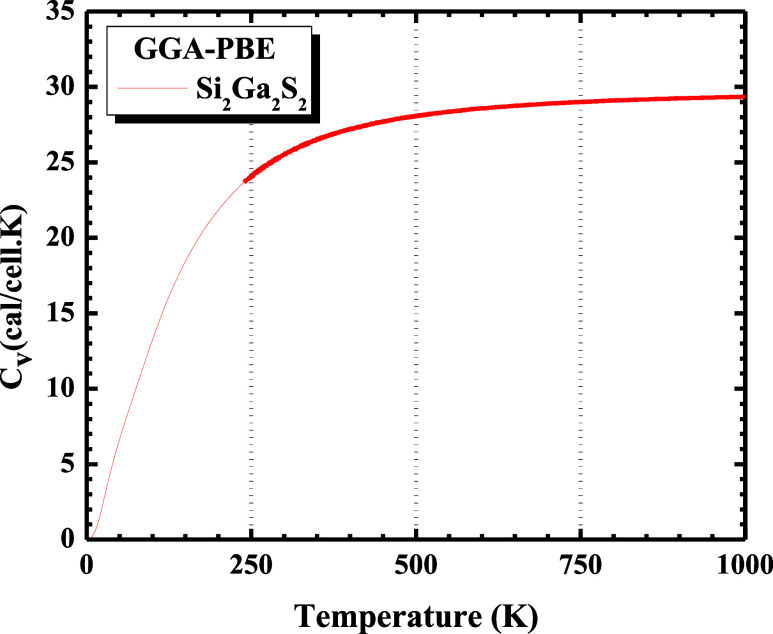
Constant volume heat capacity (*C*_V_) of the Si_2_Ga_2_S_2_ monolayer as a function of the temperature (K), using the GGA-PBE functional.

## Conclusions

In short, the 2D Si_2_Ga_2_S_2_ monolayer is investigated using first-principles calculations, focusing on the structural, electronic, optical, thermodynamic properties, energetic stability, power conversion efficiency, and the Shockley–Queisser limit in the framework of DFT. The important results can be summarized as follows: The structural parameters of the Si_2_Ga_2_S_2_ monolayer, calculated after geometry optimization, show satisfactory convergence to the symmetry of the system. The Si_2_Ga_2_S_2_ monolayer has a small direct bandgap (Γ → Γ) of 1.247 eV (2.132 eV) using the PBE (HSE06) functionals, respectively. This semiconductor characteristic also suggests the possibility of applications in specific electronic device designs such as field-effect transistors and nanosensors. The optical absorption curves (Figure 5) were displayed, proving anisotropy concerning the polarization of the incident radiation, absorbing in the UV–vis region. Moreover, due to the high absorption in UV, the Si_2_Ga_2_S_2_ monolayer could be used as a UV detector. It can also be concluded that the Si_2_Ga_2_S_2_ monolayer presented here is stable, with its stabilities proven by cohesive energy calculations and phonon-mode analysis. The power conversion efficiency, according to the Shockley–Queisser limit, could have a minimum of approximately 19.46%. The thermodynamic properties of this monolayer system are displayed by their enthalpy, entropy, free energy, and heat capacity, where free energy (*F*) indicates the possibility of a spontaneous process to obtain it.
